# Probable Seronegative Myasthenia Gravis With Initially Negative Diagnostic Testing: A Case Report

**DOI:** 10.7759/cureus.117439

**Published:** 2026-10-04

**Authors:** Sandra Rodrigues, Francisco Dias

**Affiliations:** 1 São Vicente Family Health Unit, Centro Hospitalar do Tâmega e Sousa, Penafiel, PRT; 2 União Penafidelis Family Health Unit, Centro Hospitalar do Tâmega e Sousa, Penafiel, PRT

**Keywords:** ice-pack test, left eye ptosis, muscle fatigability, myasthenia gravis (mg), ocular myasthenia gravis, seronegative myasthenia gravis

## Abstract

Myasthenia gravis (MG) is an autoimmune disorder of the neuromuscular junction characterized by fluctuating, fatigable weakness. Diagnosis can be particularly challenging when symptoms are initially non-specific and standard serological and neurophysiological investigations are unrevealing.

We report the case of a 27-year-old woman who initially developed progressive fatigue and exercise intolerance, followed by fluctuating left eyelid ptosis that worsened with exertion and improved with rest. Initial laboratory testing and cranial imaging were unrevealing. Persistence and progression of ocular symptoms prompted neurological assessment, during which an ice-pack test produced clear improvement in ptosis. Anti-acetylcholine receptor, anti-muscle-specific kinase, and anti-low-density lipoprotein receptor-related protein 4 antibodies were negative, thoracic imaging excluded thymoma, and single-fiber electromyography of the orbicularis oculi was normal, providing no objective electrophysiological confirmation of MG.

A trial of pyridostigmine provided only limited symptomatic benefit. In the context of the characteristic fluctuating phenotype, positive bedside test, exclusion of relevant alternative diagnoses, and despite the absence of objective electrophysiological confirmation, a working diagnosis of probable seronegative MG (SNMG) was established, and immunosuppressive treatment was initiated. Over the following two years, symptoms generalized, with limb fatigability, difficulty climbing stairs, occasional dysphagia, and substantial functional limitation.

This case highlights the diagnostic uncertainty inherent to suspected SNMG and the importance of longitudinal clinical assessment. Negative antibody and neurophysiological results should prompt careful reassessment and consideration of mimics rather than automatic exclusion or confirmation of MG. In primary care, recognizing true muscle fatigability, diurnal fluctuation, and evolving focal signs may facilitate timely specialist evaluation.

## Introduction

Myasthenia gravis (MG) is an autoimmune disorder of the neuromuscular junction in which impaired postsynaptic transmission produces fluctuating and fatigable skeletal-muscle weakness. Ocular manifestations, including ptosis and diplopia, are common, but early symptoms may be subtle and intermittent. The diagnosis is fundamentally clinical and is supported by antibody testing and neurophysiological studies [1,2].

Serological testing identifies pathogenic or disease-associated antibodies in most patients, particularly antibodies against the acetylcholine receptor (AChR), muscle-specific kinase (MuSK), and, in selected patients, low-density lipoprotein receptor-related protein 4 (LRP4). Nevertheless, a subgroup remains seronegative with conventional testing, although seronegativity is partly assay-dependent and more sensitive methods, including cell-based assays, may detect antibodies in some patients who test negative with conventional techniques. Recent expert consensus emphasizes that seronegative MG (SNMG) is diagnostically demanding because no single confirmatory test exists and because myopathies, congenital myasthenic syndromes, and functional neurological disorders may be mistaken for SNMG [3,4].

Ocular-predominant disease creates an additional diagnostic challenge. Repetitive nerve stimulation has a lower diagnostic yield in ocular MG than in generalized disease, while single-fiber electromyography is more sensitive but may still be negative depending on disease severity, clinical presentation, and the muscle studied [2,5]. Bedside maneuvers such as the ice-pack test can provide useful supportive evidence in patients with ptosis, although they cannot establish the diagnosis in isolation [6].

This case illustrates the longitudinal evolution from non-specific fatigue to ocular and subsequently generalized symptoms in a patient with negative conventional antibody testing and an initially non-diagnostic neurophysiological evaluation. It highlights both the value and the limits of clinical reasoning in suspected SNMG and the role of continuity of care in recognizing an evolving neuromuscular phenotype.

## Case presentation

A 27-year-old woman, previously autonomous and without relevant functional limitations, had a medical history of allergic rhinitis, vitiligo, and peripheral venous insufficiency. There was no relevant family history. She developed progressive fatigue and exercise intolerance. Initially, symptoms were most evident after usual physical activity, improved partially with rest, and were attributed to occupational workload and stress.

During the following months, the symptoms progressively worsened. She described fluctuating weakness that was more pronounced toward the end of the day and began to interfere with daily activities. She subsequently developed left unilateral eyelid ptosis. The ptosis fluctuated, worsened after exertion, and improved partially with rest.

Because of persistent symptoms, she was assessed in an acute primary-care consultation. Examination confirmed left unilateral ptosis without other focal neurological deficits or relevant systemic abnormalities. Initial laboratory investigations and cranial computed tomography did not identify an explanation for the neurological presentation.

Approximately one week later, ocular symptoms worsened (Figure 1), with visual impairment and difficulty keeping the eyes open while driving. This deterioration prompted Emergency Department assessment. Repeat cranial computed tomography was again unrevealing. Because ptosis persisted without an identified structural cause, neurological assessment was requested. An ice-pack test was performed by applying ice over the affected eyelid for approximately two minutes, after which clear clinical improvement in the left-sided ptosis was observed. The change in eyelid position was not quantitatively measured. This finding provided supportive, but not confirmatory, evidence of a neuromuscular junction disorder.

**Figure 1:**
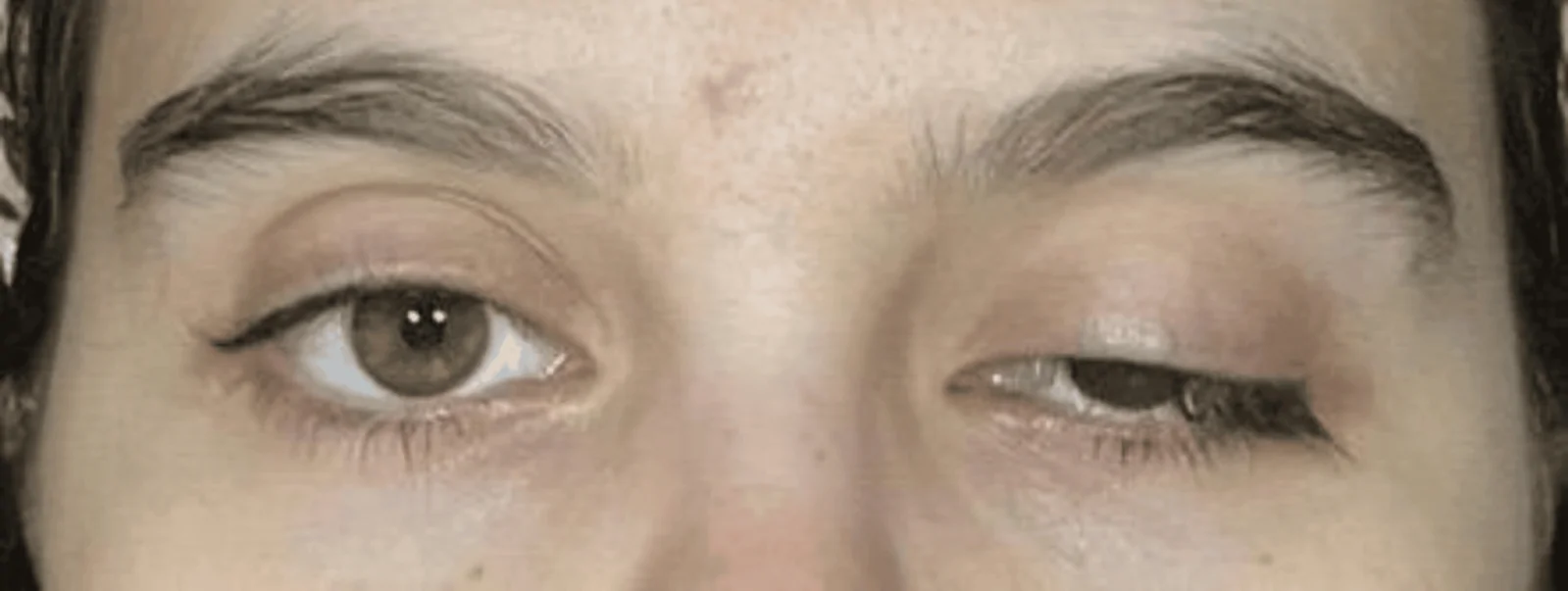
Left-sided ptosis during symptomatic presentation at the Emergency Department. The photograph demonstrates unilateral left upper eyelid ptosis prior to neurological assessment.

The patient was admitted for further etiological investigation. Cranial and orbital magnetic resonance imaging did not reveal a structural explanation for the ocular findings, and thoracic computed tomography showed no thymoma. An extended laboratory evaluation included anti-AChR, anti-MuSK, and anti-LRP4 antibodies, all of which were negative. The differential diagnostic work-up included structural, vascular, autoimmune, vasculitic, and prothrombotic causes. Cranial and orbital magnetic resonance imaging and cerebral magnetic resonance angiography showed no relevant abnormalities. Laboratory investigations were also unrevealing, including negative antinuclear and extractable nuclear antigen antibodies, anti-double-stranded DNA antibodies, PR3- and MPO-ANCA, lupus anticoagulant, anticardiolipin IgG/IgM, and anti-β2-glycoprotein I IgG/IgM antibodies, with normal complement levels. Inflammatory markers were unremarkable, and thyroid function, vitamin B12, and folate levels did not identify an alternative metabolic explanation for the clinical presentation.

Single-fiber electromyography (SFEMG) of the orbicularis oculi muscle was performed and was reported as normal. The complete original neurophysiological report, including quantitative jitter values and other technical parameters, was not available for retrospective review; therefore, objective electrophysiological confirmation of neuromuscular transmission dysfunction was not obtained.

Pyridostigmine was initially prescribed at 60 mg three times daily, with slight symptomatic improvement documented during early neurological follow-up. The dose was subsequently adjusted according to symptoms, reaching up to 60 mg six times daily (maximum 360 mg/day), although the overall symptomatic benefit remained limited. Clinical response was assessed based on the patient’s reported improvement in ocular symptoms and fatigability. The exact treatment duration could not be reliably established from the available clinical documentation.

Given the characteristic fluctuating and fatigable clinical phenotype, supportive ice-pack test, absence of an alternative diagnosis after extensive investigation, and continued neurological follow-up, the working diagnosis was probable SNMG. Prednisolone 60 mg/day was initiated. After approximately two months, slight clinical improvement was observed. High-dose corticosteroid therapy was maintained for approximately six months; attempts at dose reduction were followed by symptom recurrence, and azathioprine was subsequently introduced as a corticosteroid-sparing agent.

Over the following two years, the phenotype evolved from predominantly ocular symptoms to generalized fatigability. In addition to persistent ptosis, the patient developed marked exertional muscle fatigue, difficulty climbing stairs, limb weakness after sustained activity, occasional dysphagia, and disabling fatigue by the end of the day. These symptoms significantly affected professional functioning and activities of daily living. Treatment required progressive optimization and, at the time of the latest follow-up, consisted of prednisolone 5 mg/day and azathioprine 135 mg/day. She remained under regular Neurology and General and Family Medicine follow-up.

## Discussion

This case demonstrates a central difficulty in MG diagnosis: the phenotype may be clinically persuasive while conventional confirmatory investigations remain negative. MG diagnosis begins with recognition of fluctuating, fatigable weakness in a distribution compatible with neuromuscular junction dysfunction; laboratory and electrophysiological tests are used to support and refine that clinical impression [2]. In primary care, the distinction between non-specific tiredness and true fatigability is particularly important. A history of worsening weakness with sustained activity, improvement with rest, diurnal fluctuation, and the subsequent appearance of focal ocular signs should prompt reconsideration of common explanations for fatigue.

The term seronegative MG requires caution. The 2024 European Neuromuscular Centre workshop emphasized substantial heterogeneity in the diagnostic work-up of SNMG and highlighted the risk of misdiagnosis when objective confirmation is limited [3]. Similarly, a recent systematic review found considerable variability in how SNMG was defined across published studies, including differences in antibody methodology, electrophysiological confirmation, pharmacological testing, and response-to-treatment criteria [7]. Accordingly, the present case is best described as probable SNMG rather than unequivocally antibody-negative, electrophysiologically confirmed MG.

Seronegativity is also partly dependent on the assay used. Conventional testing may fail to detect low-affinity or low-concentration antibodies, and cell-based assays can identify antibodies in a subset of patients previously classified as seronegative [4]. In the present patient, anti-AChR, anti-MuSK, and anti-LRP4 testing was negative, but the available clinical record does not establish whether live cell-based assays were performed. This limitation is important and should be explicitly acknowledged rather than interpreting negative conventional serology as definitive evidence of a distinct seronegative subtype.

Electrophysiological testing may support the diagnosis of MG; however, its diagnostic performance depends on the technique employed, the muscles examined, and the clinical phenotype [2,5]. In the present case, single-fiber electromyography of the orbicularis oculi was performed and reported as normal. However, the complete original neurophysiological report, including quantitative jitter values and other technical parameters, was unavailable for retrospective review. Therefore, objective electrophysiological confirmation was not obtained, and the normal SFEMG result must be interpreted within the overall clinical context.

The ice-pack test was a clinically important turning point. A 2023 systematic review of 20 studies involving 1,264 participants found wide variation in sensitivity but generally high specificity in several studies, supporting the test as a useful, accessible bedside adjunct in patients with ocular features [6]. Its diagnostic value, however, is supportive rather than definitive. In this patient, the positive test gained significance because it occurred in the setting of exertion-related ptosis, partial improvement with rest, and subsequent evolution of the clinical phenotype.

The limited symptomatic improvement with pyridostigmine also illustrates why therapeutic response should not be used as a binary diagnostic test. Symptomatic response varies between patients and phenotypes, while corticosteroids and steroid-sparing immunosuppressive agents remain established components of treatment when clinically indicated [8,9]. In this case, the subsequent partial response to immunosuppression and recurrence during corticosteroid tapering were compatible with the working diagnosis but, by themselves, cannot prove it.

Longitudinal evolution provides additional context. Ocular MG may remain restricted to ocular muscles or progress to generalized disease. In the recent systematic review of SNMG, isolated ocular symptoms were common and, among patients with available longitudinal data, progression to generalized MG occurred in a substantial proportion within two years [7]. The development in this patient of limb fatigability, difficulty climbing stairs, and occasional dysphagia therefore represented a clinically meaningful evolution from an initially ocular-predominant syndrome to a generalized phenotype.

The case also illustrates the value of continuity of care. Family physicians frequently encounter fatigue, but repeated assessment allows symptom trajectories to be distinguished from isolated, non-specific complaints. Once MG is suspected or diagnosed, primary care continues to contribute through medication reconciliation, recognition of potentially aggravating drugs, monitoring for adverse effects of corticosteroids and immunosuppressants, infection prevention, vaccination review, psychosocial support, and coordination with specialist care. Contemporary international guidance supports an individualized approach to immunotherapy and emphasizes treatment goals centered on functional control with the lowest burden of adverse effects [8,9].

This report has limitations. The diagnosis lacks serological and objective electrophysiological confirmation. Although single-fiber electromyography of the orbicularis oculi was performed and reported as normal, the complete original neurophysiological report, including quantitative jitter values and other technical parameters, was unavailable for review. The antibody assay methodology was also unavailable, and no objective longitudinal MG severity scale was documented. These limitations preclude presenting the case as definitively confirmed SNMG.

## Conclusions

Fluctuating muscle fatigability can initially be mistaken for non-specific tiredness, particularly before focal neurological signs emerge. In this case, progression from exercise intolerance to exertion-related ptosis and later generalized weakness prompted a diagnostic pathway consistent with probable SNMG despite negative conventional antibody testing and an initially non-diagnostic neurophysiological study. The ice-pack test provided useful supportive evidence but was interpreted within the broader clinical context.

Suspected SNMG requires diagnostic restraint as well as persistence. Negative tests should neither automatically exclude MG nor justify the diagnosis in isolation; instead, they should lead to review of assay and electrophysiological methods, consideration of mimics, and longitudinal reassessment. For family physicians, careful characterization of fatigability, symptom fluctuation, and evolving focal signs can be decisive in identifying patients who require timely neurological evaluation.
